# Supplementary material for: Response of photosynthesis to different concentrations of heavy metals in Davidia involucrata
Source: PLoS One. 2020 Mar 16;15(3):e0228563. doi: 10.1371/journal.pone.0228563 (PMC7075629; doi:10.1371/journal.pone.0228563)
Supplement: S1 Table — (DOCX) [file pone.0228563.s001.docx]

**S1 Table. The mean and standard deviation of photosynthetic pigments of *D. involucrata* under different concentrations of Pb and Cd.**

| Treatment (mg·kg^-1^) | | Chlorophyll a (mg·g^-1^) | Chlorophyll b (mg·g^-1^) | Total chlorophyll (mg·g^-1^) | Carotenoids (mg·g^-1^) |
| --- | --- | --- | --- | --- | --- |
| Pb | 0 | 0.561 ± 0.182 | 0.324 ± 0.121 | 0.885 ± 0.303 | 0.085 ± 0.018 |
|  | 200 | 0.624 ± 0.212 | 0.317 ± 0.109 | 0.942 ± 0.321 | 0.093 ± 0.032 |
|  | 400 | 0.888 ± 0.032 | 0.477 ± 0.014 | 1.365 ± 0.047 | 0.113 ± 0.007 |
|  | 600 | 0.730 ± 0.089 | 0.364 ± 0.098 | 1.094 ± 0.187 | 0.113 ± 0.013 |
|  | 800 | 0.625 ± 0.063 | 0.285 ± 0.052 | 0.909 ± 0.107 | 0.104 ± 0.006 |
|  | 1000 | 0.844 ± 0.003 | 0.400 ± 0.020 | 1.242 ± 0.023 | 0.129 ± 0.010 |
| Cd | 0 | 0.561 ± 0.182 | 0.324 ± 0.121 | 0.885 ± 0.303 | 0.085 ± 0.018 |
|  | 1 | 0.431 ± 0.055 | 0.191 ± 0.034 | 0.948 ± 0.348 | 0.085 ± 0.007 |
|  | 5 | 0.450 ± 0.031 | 0.214 ± 0.001 | 0.627 ± 0.030 | 0.079 ± 0.006 |
|  | 10 | 0.415 ± 0.020 | 0.183 ± 0.002 | 0.562 ± 0.013 | 0.082 ± 0.004 |
|  | 20 | 0.567 ± 0.073 | 0.257 ± 0.046 | 0.781 ± 0.159 | 0.104 ± 0.015 |
|  | 30 | 0.366 ± 0.008 | 0.200 ± 0.038 | 0.694 ± 0.236 | 0.062 ± 0.024 |
| Pb+Cd | 0 | 0.561 ± 0.182 | 0.324 ± 0.121 | 0.885 ± 0.303 | 0.085 ± 0.018 |
|  | 200,1 | 0.818 ± 0.063 | 0.398 ± 0.043 | 1.216 ± 0.101 | 0.127 ± 0.003 |
|  | 400,5 | 1.224 ± 0.025 | 0.783 ± 0.095 | 2.007 ± 0.119 | 0.143 ± 0.008 |
|  | 600,10 | 1.062 ± 0.147 | 0.589 ± 0.154 | 1.651 ± 0.301 | 0.147 ± 0.008 |
|  | 800,20 | 1.073 ± 0.060 | 0.558 ± 0.039 | 1.632 ± 0.099 | 0.157 ± 0.001 |
|  | 1000,30 | 0.861 ± 0.065 | 0.433 ± 0.061 | 1.293 ± 0.122 | 0.143 ± 0.017 |
