# Supplementary material for: Response of photosynthesis to different concentrations of heavy metals in Davidia involucrata
Source: PLoS One. 2020 Mar 16;15(3):e0228563. doi: 10.1371/journal.pone.0228563 (PMC7075629; doi:10.1371/journal.pone.0228563)
Supplement: S2 Table — (DOCX) [file pone.0228563.s002.docx]

**S2 Table. The mean and standard deviation of chlorophyll synthesis and degradation products of *D. involucrata* under different concentrations of Pb and Cd.**

| Treatment (mg·kg^-1^) | | Chlorophyllase（U/L） | Mg-dechelatase（umol/L） | δ-aminolevulinic acid（U/L） | Porphobilinogen (ug/mL) | Uroporphyrinogen (ug/mL) |
| --- | --- | --- | --- | --- | --- | --- |
| Pb | 0 | 0.923 ± 0.032 | 0.041 ± 0.002 | 217.242 ± 46.246 | 1.038 ± 0.233 | 0.144 ± 0.062 |
|  | 200 | 0.558 ± 0.027 | 0.305 ± 0.094 | 1942.407 ± 199.348 | 0.571 ± 0.129 | 0.243 ± 0.004 |
|  | 400 | 2.119 ± 0.017 | 0.039 ± 0.003 | 188.882 ± 3.305 | 0.216 ± 0.036 | 0.184 ± 0.014 |
|  | 600 | 0.776 ± 0.122 | 0.038 ± 0.007 | 185.150 ± 9.956 | 0.355 ± 0.151 | 0.200 ± 0.011 |
|  | 800 | 0.713 ± 0.041 | 0.052 ± 0.015 | 579.780 ± 209.618 | 0.992 ± 0.241 | 0.057 ± 0.003 |
|  | 1000 | 1.042 ± 0.044 | 0.345 ± 0.008 | 1378.311 ± 46.086 | 0.563 ± 0.111 | 0.219 ± 0.047 |
| Cd | 0 | 0.923 ± 0.032 | 0.041 ± 0.002 | 353.582 ± 146.568 | 1.038 ± 0.233 | 0.144 ± 0.062 |
|  | 1 | 0.301 ± 0.054 | 0.043 ± 0.008 | 202.087 ± 29.093 | 0.252 ± 0.051 | 0.178 ± 0.007 |
|  | 5 | 0.718 ± 0.088 | 0.038 ± 0.005 | 205.395 ± 27.237 | 0.776 ± 0.146 | 0.220 ± 0.010 |
|  | 10 | 0.783 ± 0.010 | 0.038 ± 0.000 | 381.490 ± 70.717 | 0.967 ± 0.059 | 0.195 ± 0.004 |
|  | 20 | 0.825 ± 0.024 | 0.275 ± 0.070 | 1282.984 ± 1.349 | 0.524 ± 0.114 | 0.077 ± 0.025 |
|  | 30 | 0.654 ± 0.144 | 0.035 ± 0.005 | 183.125 ± 62.570 | 0.156 ± 0.021 | 0.009 ± 0.001 |
| Pb+Cd | 0 | 0.923 ± 0.032 | 0.054 ± 0.022 | 217.242 ± 46.246 | 0.725 ± 0.209 | 0.144 ± 0.062 |
|  | 200,1 | 1.354 ± 0.124 | 0.028 ± 0.000 | 146.607 ± 10.497 | 0.168 ± 0.015 | 0.219 ± 0.010 |
|  | 400,5 | 0.358 ± 0.154 | 0.027 ± 0.003 | 154.407 ± 0.535 | 0.169 ± 0.011 | 0.191 ± 0.017 |
|  | 600,10 | 0.192 ± 0.007 | 0.023 ± 0.002 | 155.202 ± 8.039 | 0.177 ± 0.028 | 0.194 ± 0.031 |
|  | 800,20 | 0.725 ± 0.039 | 0.039 ± 0.015 | 175.022 ± 40.281 | 0.314 ± 0.113 | 0.237 ± 0.005 |
|  | 1000,30 | 0.940 ± 0.066 | 0.115 ± 0.020 | 431.572 ±148.944 | 0.582 ± 0.169 | 0.238 ± 0.017 |
