# Supplementary material for: Response of photosynthesis to different concentrations of heavy metals in Davidia involucrata
Source: PLoS One. 2020 Mar 16;15(3):e0228563. doi: 10.1371/journal.pone.0228563 (PMC7075629; doi:10.1371/journal.pone.0228563)
Supplement: S3 Table — (DOCX) [file pone.0228563.s003.docx]

**S3 Table****. The mean and standard deviation of gas-exchange parameters of *D. involucrata* under different concentrations of Pb and Cd.**

| Treatment (mg·kg^-1^) | | Net photosynthetic rate (umol·m^-2^·s^-1^) | Intercellular CO_2_ concentration (umol·m^-2^·s^-1^) | Stomatal conductance (umol·m^-2^·s^-1^) | Transpiration rate (umol·m^-2^·s^-1^) |
| --- | --- | --- | --- | --- | --- |
| Pb | 0 | 3.927 ± 0.928 | 0.041 ± 0.018 | 244.353 ± 37.430 | 0.840 ± 0.271 |
|  | 200 | 5.102 ± 0.960 | 0.052 ± 0.014 | 254.207 ± 33.240 | 1.058 ± 0.279 |
|  | 400 | 4.483 ± 0.681 | 0.049 ± 0.012 | 258.134 ± 33.129 | 1.024 ± 0.224 |
|  | 600 | 3.822 ± 0.935 | 0.036 ± 0.009 | 232.961 ± 26.732 | 0.914 ± 0.209 |
|  | 800 | 2.861 ± 1.180 | 0.036 ± 0.010 | 282.518 ± 40.510 | 0.864 ± 0.220 |
|  | 1000 | 1.559 ± 0.989 | 0.018 ± 0.008 | 273.499 ± 57.506 | 0.503 ± 0.210 |
| Cd | 0 | 3.927 ± 0.928 | 0.041 ± 0.018 | 244.353 ± 37.430 | 0.840 ± 0.271 |
|  | 1 | 2.794 ± 0.436 | 0.034 ± 0.008 | 256.078 ± 28.698 | 1.135 ± 0.182 |
|  | 5 | 2.186 ± 0.635 | 0.030 ± 0.010 | 267.976 ± 24.178 | 1.085 ± 0.330 |
|  | 10 | 1.872 ± 0.476 | 0.033 ± 0.009 | 338.253 ± 21.663 | 0.509 ± 0.225 |
|  | 20 | 2.674 ± 0.563 | 0.042 ± 0.006 | 313.799 ± 18.279 | 0.852 ± 0.116 |
|  | 30 | 2.183 ± 0.915 | 0.033 ± 0.008 | 300.009 ± 22.447 | 0.756 ± 0.171 |
| Pb+Cd | 0 | 3.927 ± 0.928 | 0.041 ± 0.018 | 244.353 ± 37.430 | 0.840 ± 0.271 |
|  | 200,1 | 3.121 ± 0.902 | 0.035 ± 0.010 | 248.087 ± 14.704 | 0.928 ± 0.226 |
|  | 400,5 | 1.645 ± 0.930 | 0.024 ± 0.006 | 290.389 ± 47.649 | 0.736 ± 0.175 |
|  | 600,10 | 2.107 ± 0.838 | 0.026 ± 0.007 | 260.671 ± 26.600 | 0.888 ± 0.257 |
|  | 800,20 | 2.832 ± 1.055 | 0.045 ± 0.016 | 277.120 ± 28.110 | 1.538 ± 0.516 |
|  | 1000,30 | 1.200 ± 0.930 | 0.031 ± 0.016 | 267.945 ± 32.406 | 1.116 ± 0.486 |
