# Supplementary material for: Response of photosynthesis to different concentrations of heavy metals in Davidia involucrata
Source: PLoS One. 2020 Mar 16;15(3):e0228563. doi: 10.1371/journal.pone.0228563 (PMC7075629; doi:10.1371/journal.pone.0228563)
Supplement: S4 Table — (DOCX) [file pone.0228563.s004.docx]

**S4 Table. The mean and standard deviation of accumulation factors(BFC) of *D. involucrata* under different concentrations of Pb and Cd.**

| Treatment (mg·kg^-1^) | | root | stem | leaf |
| --- | --- | --- | --- | --- |
| Pb | 0 | 5.839 ± 0.320 | 0.036 ± 0.013 | 0.156 ± 0.005 |
|  | 200 | 1.886 ± 1.250 | 0.016 ± 0.007 | 0.038 ± 0.013 |
|  | 400 | 0.682 ± 0.297 | 0.016 ± 0.007 | 0.015 ± 0.002 |
|  | 600 | 0.542 ± 0.241 | 0.006 ± 0.001 | 0.005 ± 0.001 |
|  | 800 | 0.919 ± 0.159 | 0.010 ± 0.002 | 0.004 ± 0.000 |
|  | 1000 | 1.204 ± 0.344 | 0.013 ± 0.001 | 0.006 ± 0.001 |
| Cd | 0 | 18.628 ± 0.835 | 0.412 ± 0.128 | 8.193 ± 1.977 |
|  | 1 | 3.043 ± 1.245 | 0.315 ± 0.153 | 0.704 ± 0.426 |
|  | 5 | 4.715 ± 3.235 | 0.013 ± 0.005 | 0.016 ± 0.003 |
|  | 10 | 4.641 ± 0.267 | 0.005 ± 0.003 | 0.015 ± 0.003 |
|  | 20 | 3.894 ± 2.382 | 0.005 ± 0.000 | 0.005 ± 0.002 |
|  | 30 | 1.709 ± 0.699 | 0.002 ± 0.000 | 0.002 ± 0.001 |
| Pb+Cd | 0 | 5.839 ± 0.320 | 0.036 ± 0.013 | 0.156 ± 0.005 |
|  | 200,1 | 1.128 ± 0.336 | 0.002 ± 0.000 | 0.006 ± 0.003 |
|  | 400,5 | 1.502 ± 0.659 | 0.001 ± 0.001 | 0.001 ± 0.000 |
|  | 600,10 | 1.338 ± 0.202 | 0.001 ± 0.001 | 0.001 ± 0.000 |
|  | 800,20 | 0.822 ±0.075 | 0.001 ± 0.000 | 0.003 ± 0.000 |
|  | 1000,30 | 0.546 ± 0.002 | 0.001 ± 0.000 | 0.003 ± 0.001 |
